# Aflatoxin exposure and health impacts: global burden and advances in detection technologies

**DOI:** 10.1186/s41021-026-00363-1

**Published:** 2026-07-31

**Authors:** Gudisa Bereda

**Affiliations:** Department of Pharmacy, Marie Stopes International Ethiopia (MSIE), Ambo, Ethiopia

**Keywords:** Aflatoxin exposure, Health impacts, Global burden, Aflatoxins, Food contaminants, Hepatocellular carcinoma, Immune suppression, Detection technologies

## Abstract

**Background and aims:**

Aflatoxins are food contaminants that cause exposure and adverse health effects and are detected using modern analytical methods. This review summarizes global aflatoxin exposure levels, exposure routes, associated health effects, and detection technologies.

**Methods:**

This narrative review was guided by the Scale for the Assessment of Narrative Review Articles (SANRA) framework. Relevant literature was retrieved from PubMed, Scopus, Web of Science, and Google Scholar. Studies addressing aflatoxin exposure, health effects, and detection technologies were included and qualitatively synthesized.

**Results:**

This review showed that in Côte d’Ivoire, peanut paste had 100% AFB1 contamination, with levels of 4535 µg/kg (AFB1) and 8094 µg/kg (total aflatoxins), and 99% of samples exceeded European Union limits. Dietary intake is the main route of exposure (0.3–180 ng/kg/day), with additional exposure via prenatal transfer, breastfeeding (0.01–0.55 ng/mL AFM1), and complementary feeding. Biomarkers show high aflatoxin exposure: 97% (Malaysia) and 94% (pregnant women in Nepal; 0.45–2939.30 pg/mg). Aflatoxin exposure is responsible for 4.6–28.2% of global hepatocellular carcinoma cases, contributes to approximately 250,000 deaths annually, and is associated with a 2–4-fold higher risk of cancer in children. High-performance liquid chromatography coupled with fluorescence detection (HPLC-FLD) is widely used, while Liquid chromatography–tandem mass spectrometry (LC–MS/MS) is the gold standard (< 1 ng/mL). Electrochemical immunosensors reach 0.3 pg/mL, and lateral flow assays provide rapid but less sensitive screening.

**Conclusion:**

Aflatoxin exposure is a major global public health concern. Improved detection methods and food safety measures are essential to reduce exposure and disease burden.

**Supplementary Information:**

The online version contains supplementary material available at 10.1186/s41021-026-00363-1.

## Introduction

Aflatoxins were first recognized in the United Kingdom in 1960 following the outbreak known as “turkey X disease,” which caused the death of over 100,000 turkeys and other poultry species [1]. The toxic compound was later named “aflatoxin,” derived from its fungal source *Aspergillus flavus* (“A” for *Aspergillus* and “fla” for *flavus*) [1]. Since then, aflatoxins have become a major global food safety concern. It is estimated that nearly 4.5 billion individuals worldwide consume food products contaminated with aflatoxin residues [2]. More than 5 billion people in low-income regions experience continuous exposure due to their reliance on vulnerable staple crops [3]. According to the Food and Agriculture Organization (FAO), about 25% of global agricultural commodities are contaminated with mycotoxins during pre-harvest, post-harvest, or storage due to favorable fungal conditions [4]. These toxins resist processing and cooking, remaining stable despite conventional decontamination methods.

Aflatoxins are primarily produced by *Aspergillus flavus* and *Aspergillus parasiticus* [5]. More than 20 structural variants have been identified. *A. flavus* mainly produces AFB1 and AFB2, whereas *A. parasiticus* produces AFB1, AFB2, AFG1, and AFG2 [6]. The most significant forms are AFB1, AFB2, AFG1, and AFG2 [7]. Their toxicity ranking is: AFB1 = AFM1 > AFG1 > AFB2 = AFM2 > AFG2 [8]. Among them, AFB1 is the most prevalent and is considered one of the most potent naturally occurring genotoxic and carcinogenic compounds [6–8]. Human exposure occurs mainly through ingestion of contaminated food, although inhalation, dermal absorption, and transplacental transfer have also been reported [9]. Frequently contaminated staples include maize, peanuts, rice, sorghum, wheat, pearl millet, and dairy products containing AFM1 [7]. Due to their toxicity, strict regulatory limits have been established. For instance, the European Pharmacopoeia sets maximum limits of 2 µg/kg for AFB1 and 4 µg/kg for total aflatoxins in herbal medicinal products [10].

Aflatoxins are highly substituted difuranocoumarin compounds with strong teratogenic, mutagenic, immunosuppressive, and hepatotoxic properties in humans and animals [11]. AFB1 is classified as a Group 1 human carcinogen by the International Agency for Research on Cancer (IARC) [12]. Immunocompromised individuals, including patients with HIV/AIDS, malignancies, diabetes, chronic hepatitis, autoimmune diseases, or organ transplant recipients, are particularly vulnerable [13]. Chronic AFB1 exposure significantly increases the risk of hepatocellular carcinoma (HCC) and liver cirrhosis, especially in individuals infected with the hepatitis B virus (HBV) [14]. Given the widespread contamination of food commodities and the serious toxicological consequences of aflatoxin exposure, there is a critical need for analytical methods that are highly sensitive, rapid, and affordable [1]. These methods should also be capable of detecting trace levels in complex food and herbal matrices.

This review integrates synthesis of aflatoxin exposure, biomonitoring evidence, health effects, and detection technologies into a unified framework rather than a descriptive review. It connects exposure pathways, biomarkers, and health outcomes in a continuous exposure biomarker pathway across populations and life stages. The review further provides a comparative global synthesis of contamination levels, biomarker prevalence, and regulatory exceedances across countries and commodities, highlighting exposure disparities and vulnerable groups. In addition, it critically addresses methodological variability in biomonitoring due to differences in biological matrices, metabolism, and analytical platforms, which affects cross-study comparability. It synthesizes available evidence on detection methods. These include confirmatory techniques (LC–MS/MS and UHPLC–MS/MS), intermediate biosensors (electrochemical immunosensors, LSPR, SERS), and rapid screening tools (ELISA, LFAs, and paper-based assays). This synthesis highlights their complementary roles in surveillance systems.

## Methods

This integrative review synthesized evidence on aflatoxin prevalence, exposure pathways, health effects, and emerging detection technologies. The review was designed as a narrative integrative synthesis rather than a systematic review or meta-analysis. Therefore, PRISMA guidelines were not applied. A comprehensive literature search was conducted in PubMed, Scopus, Web of Science, and Google Scholar. The search covered studies published between January 2000 and January 2026 to capture both foundational and recent evidence on aflatoxin research, exposure, toxicology, and detection technologies. A structured combination of Boolean search terms was used, including “aflatoxin prevalence,” “aflatoxin exposure pathways,” “dietary aflatoxin exposure,” “biomonitoring aflatoxin,” “AFB1,” “AFM1,” “health effects,” “hepatotoxicity,” “hepatocellular carcinoma,” “aflatoxin detection methods,” “biosensors,” “nanotechnology,” “LC–MS/MS,” “immunoassay,” “molecular detection,” and “emerging technologies.” Reference lists of eligible articles and relevant reviews were manually screened to identify additional studies. Studies were included if they were peer-reviewed original research articles (experimental, clinical, epidemiological, or analytical studies), systematic reviews, meta-analyses, surveillance reports, or international guidelines. Eligible studies addressed aflatoxin prevalence, exposure pathways, toxicological effects, or detection. Non-English publications without available translations, conference abstracts without full data, opinion pieces, editorials, and studies with insufficient methodological detail were excluded.

Study selection involved screening of titles, abstracts, and full texts. Duplicates were removed before screening. Data were extracted using a standardized framework capturing study design, population or sample type, exposure pathways, health outcomes, and analytical or detection methods. Any disagreements were resolved through discussion with an external reviewer and careful re-assessment against the predefined eligibility criteria. Findings were synthesized thematically into four domains: (1) global and regional prevalence patterns; (2) exposure pathways and risk determinants; (3) toxicological and clinical health effects; and (4) conventional and emerging detection technologies. The methodological rigor of included studies was assessed using the Scale for the Assessment of Narrative Review Articles (SANRA) tool [15]. SANRA evaluates clarity of aims, literature search transparency, referencing quality, scientific reasoning, and presentation of evidence (see Supplementary Table 1 for SANRA assessment). This tool was selected because the review follows a narrative integrative design rather than a systematic review framework.

## Global and regional prevalence of aflatoxins

The FAO previously estimated that approximately one-fourth of global food crops were contaminated with mycotoxins before 1985 [4]. Contamination levels above European Union (EU) and Codex limits are consistent with this estimate, while contamination at detectable levels is considerably higher, reaching 60–80% [16]. Aflatoxin contamination in food remains a major global food safety problem, particularly in warm and humid regions that favor fungal growth and toxin production [17]. The prevalence and levels of contamination vary depending on crop type, storage conditions, farming practices, climate factors, and the strength of regulatory systems [18]. Commonly affected foods include maize, peanuts, rice, sorghum, spices, milk, and animal feed [19]. The problem is more severe in low- and middle-income countries due to poor post-harvest drying, inadequate storage infrastructure, and weak surveillance systems [20].

### Food commodities (dietary contamination)

Maize, a primary dietary staple in Nepal, has been identified as a major source of aflatoxin contamination in maize-derived food products [21] (Table 1). Although rice is the dominant staple in Nepal and Bangladesh, it generally exhibits low levels of aflatoxin contamination [22]. In contrast, other commonly consumed foods in Bangladesh, including betel nut, lentils, and red chili powder, have been reported to contain aflatoxin concentrations [23]. This highlights that aflatoxin exposure risk in South Asia is not uniform across staple foods but is strongly influenced by dietary diversification, food processing practices, and regional storage conditions.

Manizan et al. (2018) reported that in Côte d’Ivoire, all peanut paste samples were contaminated with aflatoxin B1. They found that 99% exceeded EU limits of 2 µg/kg for AFB1 and 4 µg/kg for total aflatoxins. The highest concentrations reached 4535 µg/kg for AFB1 and 8094 µg/kg for total aflatoxins [24]. Regarding other commodities, maize samples showed 96% aflatoxin B1, whereas rice samples showed 57% contamination. In maize, 58% exceeded EU limits. In rice, 24% exceeded EU limits. The maximum levels were 80 µg/kg in maize and 14 µg/kg in rice [24]. This indicates that peanut paste and maize carried considerably greater aflatoxin risk than rice. Mwalwayo and Thole (2016) reported that maize samples in Malawi were analyzed for aflatoxins. The detection limits were 2 µg/kg for aflatoxins [25]. About 20% of samples exceeded national aflatoxin limits. The maximum aflatoxin level was 140 µg/kg [25]. These findings indicate notable maize contamination and ongoing risks of dietary aflatoxin exposure despite sensitive detection methods.

**Table 1:** Aflatoxin contamination in food commodities

Country	Food commodity/matrix	Mycotoxins detected	Contamination prevalence (%)	Maximum concentration	Regulatory exceedance	Findings	Reference
Nepal	Maize and maize-derived food products	Aflatoxins	Not reported	Not reported	Not reported	Maize is identified as a major source of aflatoxin contamination	[21]
	Rice	Aflatoxins	Not reported	Not reported	Not reported	Generally, low aflatoxin contamination levels are reported	[22]
Bangladesh	Rice	Aflatoxins	Not reported	Not reported	Not reported	Generally, low aflatoxin contamination levels are reported	[22]
	Betel nut, lentils, and red chili powder	Aflatoxins	Not reported	Not reported	Not reported	Substantial aflatoxin concentrations detected	[23]
Côte d’Ivoire	Peanut paste	AFB1, total aflatoxins, co-occurring mycotoxins	100% contaminated with AFB1	AFB1: 4535 µg/kg; total aflatoxins: 8094 µg/kg	99% exceeded EU limits	High multi-mycotoxin diversity with 15 co-occurring toxins	[24]
	Maize	AFB1	96% contaminated with AFB1	AFB1: 80 µg/kg	58% exceeded EU limits	Aflatoxin in 58% of samples	
Malawi	Maize	Aflatoxins	Not reported	Aflatoxins: 140 µg/kg	About 20% exceeded national aflatoxin limits	Aflatoxin contamination toxins above 140 µg/kg observed	[25]
	Regional maize samples	Aflatoxins	Not reported	Not reported	Not reported	Higher contamination levels are reported in the southern region than in the northern region.	

### Biomonitoring studies (human exposure)

Human exposure studies across Asia, Africa, and the Middle East consistently detect aflatoxin biomarkers in biological samples, indicating pervasive dietary exposure [11] (Table 2). In Asia, high exposure has been documented in multiple populations. In Malaysia, AFB1–lysine adducts were detected in 97% of serum samples [26]. In Nepal and Bangladesh, isotope-dilution mass spectrometry revealed very high aflatoxin exposure. Biomarkers were detected in 94% of pregnant women in Nepal and in all Bangladeshi mothers, cord blood samples, and infants, confirming transplacental and early-life exposure [27]. This suggests that aflatoxin exposure in South Asia is not only widespread but begins in utero and continues into infancy, raising concerns about early developmental toxicity and intergenerational health effects.

In East Africa, similarly high exposure levels were observed. In Kenya, AF-alb adducts were detected in 78% of serum samples [28], while in Uganda, 98% of participants showed detectable levels [29]. In Tanzania, detection rates among young children ranged from moderate to very high (67–99%) [30]. These consistently high biomarker prevalence rates across East African countries indicate sustained dietary exposure and suggest a persistent failure of food safety control systems across vulnerable rural and peri-urban populations.

In North Africa, exposure patterns were more variable but still substantial. In Egypt, AF-alb was detected in 67% of samples in one study [31], while another reported a lower prevalence of 35.6% in pregnant women [32]. However, in South Africa, AFM1 was not detected in urine samples, suggesting possible regional differences in exposure or dietary intake patterns [33]. In South Africa, all dairy farm milk samples contained AFM1 at concentrations of 0.02–1.5 µg/L, while retail milk samples showed contamination levels ranging from 0.01 to 3.1 µg/L [34]. AFM1 levels above 0.05 µg/L were detected in 90.6% and 62.1% of cattle milk samples, and in 76% and 53.8% of goat milk samples from Mapate and Nwanedi, respectively [35]. In maize samples from rural northern South Africa, AFB1 concentrations ranged from 1 to 133 µg/kg in village-derived maize but remained below 1.0 µg/kg in genetically sorted maize [36]. In contrast, AFM1 was not detected in urine samples in South Africa, suggesting potential differences in exposure levels, dietary intake, or toxin metabolism between food and human biological samples. These variations highlight important knowledge gaps, particularly regarding regional dietary habits, food monitoring coverage, and biomarker sensitivity, which may affect true exposure in some settings.

The comparative evidence reveals three key gaps: (i) inconsistent exposure surveillance across regions, (ii) limited longitudinal data linking biomarker exposure to long-term health outcomes, and (iii) insufficient integration of dietary, environmental, and socioeconomic determinants of exposure. From a public health perspective, these findings underscore the urgent need for harmonized biomonitoring systems and strengthened food safety regulations. Targeted interventions are also needed for high-risk populations such as pregnant women and young children, where exposure may lead to irreversible developmental consequences.

**Table 2:** Aflatoxin biomarker detection and human exposure studies

Country	Population/matrix	Biomarker	Detection (%)	Concentration range	Findings	Reference
Malaysia	Serum	AFB1–lysine adduct	97%	0.20–23.26 pg/mg	High exposure detected in serum samples	[26]
Nepal	Pregnant women’s serum	AFB1–lysine adduct	94%	0.45–2939.30 pg/mg	High exposure among pregnant women	[27]
Bangladesh	Mothers, cord blood, and infants	AFB1–lysine adduct	100%	Not reported	Universal detection reported	[27]
Kenya	Serum	AF-alb adduct	78%	0–211 pg/mg	Detectable serum biomarker levels observed	[28]
Uganda	Serum	AF-alb adduct	98%	0–237.7 pg/mg	Very high biomarker detection prevalence	[29]
Tanzania	Young children	Aflatoxin biomarkers	67–99%	Not reported	Moderate to very high detection rates reported	[30]
Egypt	Serum	AF-alb adduct	67%	0–32.8 pg/mg	Detectable aflatoxin exposure observed	[31]
	Pregnant women	AF-alb adduct	35.6%	Not reported	Lower prevalence reported in pregnant women	[32]
South Africa	Urine	AFM1	0%	Not detected	AFM1 not detected in urine samples	[33]
	Dairy farm milk	AFM1	Reported in all samples	0.02–1.5 µg/L	All dairy farm milk samples contaminated	[34]
	Retail milk	AFM1	Reported in all samples	0.01–3.1 µg/L	Retail milk contamination observed	[34]
	Cattle milk	AFM1	62.1–90.6% above 0.05 µg/L	> 0.05 µg/L	High prevalence above EU limits	[35]
	Goat milk	AFM1	53.8–76% above 0.05 µg/L	> 0.05 µg/L	High prevalence above EU limits	[35]
	Rural maize samples	AFB1	Not reported	AFB1: 1–133 µg/kg	Higher contamination in village-derived maize than in genetically sorted maize	[36]

## Exposure pathways of aflatoxin in humans

**Fig. 1:**
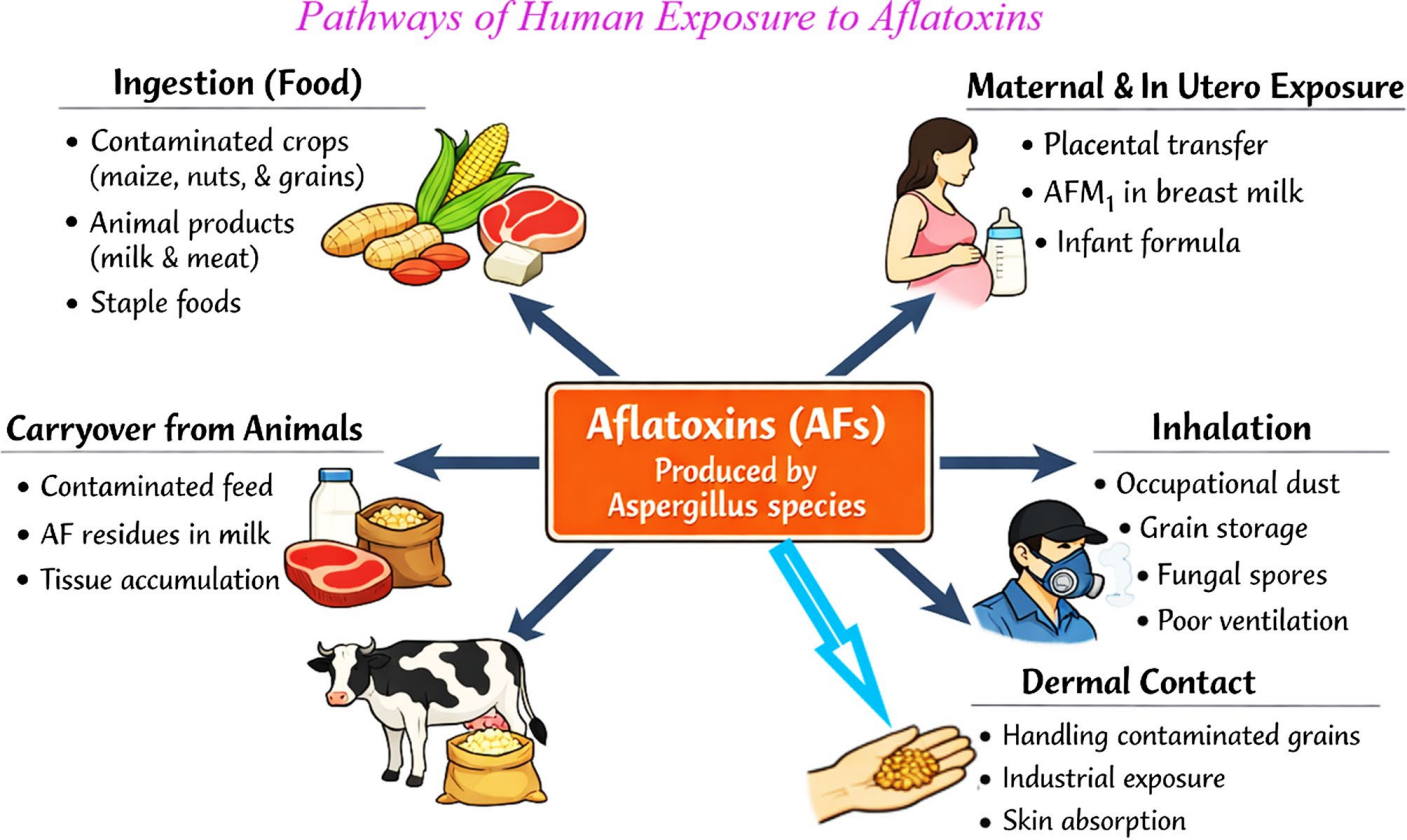
Pathways of human exposure to aflatoxins

Human exposure to aflatoxins can occur through several pathways, including ingestion of contaminated food, consumption of animal products such as milk, meat, and egg residues, and inhalation of contaminated dust particles in the environment [37–39] (Table 3; Fig. 1). Additional exposure pathways include prenatal and maternal transfer, dermal contact, and inhalation exposure. In tropical regions, environmental conditions and climatic fluctuations, combined with traditional farming practices, increase crop susceptibility to fungal invasion [40], thereby promoting the production of aflatoxins. These conditions contribute indirectly to human exposure by increasing contamination of food and environmental media.

### Exposure to AF through ingestion

Aflatoxin exposure represents a major global health concern, particularly in low-resource settings where food handling systems, storage infrastructure, and quality control measures are inadequate [37]. Exposure pathways include ingestion of contaminated food, which remains the primary route. They also include consumption of animal products such as milk, meat, and eggs with aflatoxin residues, as well as inhalation of contaminated dust in environmental settings. Additional pathways include dermal contact and maternal transfer during pregnancy and lactation [39]. According to Kibugu et al. (2024) [41], exposure assessment focuses on the extent, frequency, and dietary sources through which aflatoxins enter different population groups, emphasizing how and from where exposure occurs. This highlights that aflatoxin exposure is driven by multiple interconnected pathways involving dietary intake, environmental contact, and maternal–infant transfer.

Foodborne exposure remains a major source of aflatoxin exposure. This is especially important in regions where staple crops are highly susceptible to fungal contamination. The World Health Organization (WHO) estimated in 2015 that unsafe food causes approximately 600 million illnesses annually worldwide [42]. This results in about 420,000 deaths and the loss of 33 million healthy life years. A substantial proportion of this disease burden is attributed to exposure to aflatoxins through the consumption of contaminated food. Aflatoxins are primarily synthesized by *Aspergillus* species, particularly *A. flavus* and *A. parasiticus*, whereas *Penicillium* and *Fusarium* are responsible for producing other major mycotoxins rather than aflatoxins [43].

Aflatoxin exposure primarily occurs through dietary ingestion, and the Joint FAO/WHO Expert Committee on Food Additives estimated mean daily intake levels of 0.93–2.45 ng/kg body weight in Europe, 3.5–180 ng/kg body weight in Africa, and 0.3–53 ng/kg body weight in Asia [26]. Alberts et al. (2017) [44] reported that in some rural populations, high maize consumption of 1–2 kg per person per day further increases dietary exposure to aflatoxins. These data indicate regional variation in exposure pathways through contaminated staple food consumption, with higher dietary intake levels in Africa, intermediate levels in Asia, and lower levels in Europe. This variation reflects differences in food consumption patterns, agricultural practices, food storage conditions, and regulatory enforcement, all of which influence the extent of dietary aflatoxin exposure across populations.

### In utero and maternal exposure

Aflatoxin exposure may begin before birth, as aflatoxins can cross the placental barrier [45]. Prenatal exposure occurs through maternal dietary intake of contaminated foods, leading to transfer of aflatoxins to the fetus during pregnancy [45]. Hernandez-Vargas et al. (2015) assessed prenatal exposure in infants aged 2–8 months using aflatoxin–albumin (AF-alb) adducts as a biomarker of maternal dietary intake during gestation, and related this to genome-wide DNA methylation patterns in peripheral blood cells [46].

Both low-dose and high-dose exposure groups assessed internal aflatoxin exposure following maternal administration, with aflatoxin exposure in the offspring confirmed after birth [47]. Postnatal exposure may also continue depending on maternal intake and exposure status during lactation periods [47].

Breast milk represents another important source of aflatoxin exposure. The metabolite aflatoxin M1 (AFM1) can appear in breast milk within 12–24 h after maternal intake of contaminated foods [29]. Studies conducted by Magoha et al. (2016) [48] and Mahdavi et al. (2010) [49] consistently demonstrate widespread detection of AFM1 in breast milk, indicating continuous maternal dietary intake is a source of infant exposure through lactation. Elevated levels of aflatoxins have also been documented in maternal milk samples from Italy [50]. Infant formula feeding may represent an additional dietary exposure pathway for aflatoxins in early life [48]. An epidemiological study from Iran reported detectable levels of AFM1 in breast milk, indicating infant exposure through maternal diet [51]. Similarly, Magoha et al. (2014) [52] evaluated AFM1 concentrations in breast milk from 143 nursing mothers in Northern Tanzania. They reported measurable levels of AFM1 in breast milk (0.01–0.55 ng/mL). Infant exposure during the first six months of life was also estimated, with mean intakes of 11.08 ± 10.13, 11.94 ± 9.69, and 10.91 ± 6.82 ng/kg body weight at months 1, 3, and 5, respectively. These findings demonstrate that aflatoxin exposure can occur during exclusive breastfeeding, highlighting breastfeeding as a continuous early-life exposure route with measurable intake levels across infancy.

Intake of contaminated foods during pregnancy and lactation represents a major aflatoxin exposure pathway [53]. This is particularly important during the first 1,000 days from conception to two years of age, a highly sensitive period for growth and development. In Egypt, 36% of maternal blood samples contained AF-alb adducts, indicating measurable dietary exposure in the population [54]. In West African populations, over 95% of blood samples tested positive for AF-alb, reflecting sustained exposure through contaminated food consumption [54]. These differences suggest regional variation in exposure pathways. Dietary patterns, food storage practices, agricultural methods, and regulatory enforcement between North and West Africa likely influence them.

### Exposure via weaning foods

Young children beginning complementary feeding are particularly vulnerable to aflatoxin exposure because staple weaning foods such as maize and groundnuts are frequently contaminated with fungi [55]. Compared with adults, children experience higher aflatoxin exposure per body weight, increasing their vulnerability due to rapid growth and higher nutritional needs [56]. Early-life exposure occurs mainly through contaminated complementary foods, especially cereal- and maize-based diets commonly introduced during infancy. Additional exposure occurs via maternal–infant transfer, including prenatal placental transfer and postnatal exposure through breast milk containing aflatoxin metabolites [56, 57]. Higher food intake per body weight further increases internal exposure during early childhood [57].

In a cross-sectional study involving 480 children aged 9 months to 5 years in Benin and Togo, aflatoxin biomarkers increased after the introduction of complementary foods and peaked around three years of age [58]. They showed significant inverse associations with HAZ, WAZ, and WHZ scores after adjustment for confounding variables. Similarly, Okoth and Ohingo (2004) reported higher exposure through consumption of aflatoxin-contaminated weaning flour among 53.8% of wasted children compared with 27.7% of non-wasted children in a study of 242 children aged 3–36 months [59]. This finding supports the aforementioned cross-sectional study by indicating that aflatoxin exposure during the complementary feeding period is associated with poor growth outcomes, particularly wasting. It is also consistent with the inverse relationship reported between aflatoxin exposure and anthropometric indices (HAZ, WAZ, and WHZ).

In Egypt, a study involving 46 children aged 1 month to 4.5 years found detectable aflatoxin exposure, indicating early-life dietary intake as an important exposure pathway [60]. Turner et al. (2003) assessed aflatoxin exposure in 466 Gambian children aged 6–9 years using serum AF-alb adducts as a biomarker of dietary intake from contaminated foods [61]. Likewise, a Kenyan study involving 199 children aged 6–17 years evaluated exposure through blood-based biomarker detection, indicating ongoing dietary exposure in the population [62]. These results extend the evidence to older children, confirming that aflatoxin exposure persists beyond early childhood through continued dietary intake of contaminated foods. Biomarker-based detection indicates ongoing exposure across age groups, reflecting sustained ingestion of aflatoxin-contaminated diets during childhood development.

### Exposure through carryover from animal sources

Aflatoxins originate in crops contaminated by Aspergillus fungi under warm, humid conditions before harvest and during post-harvest storage due to poor drying and handling [63]. These contaminated cereals and nuts are consumed directly by humans or used as animal feed [64]. Livestock then consume feed contaminated with aflatoxins derived from fungal-infected crops [64]. In ruminants, rumen microbiota can partially degrade aflatoxins and reduce their bioavailability, whereas monogastric animals lack this detoxification capacity and are more susceptible to toxicity [65]. After ingestion, aflatoxins are absorbed in the gastrointestinal tract of the animal and transported to the liver [66]. In the liver, aflatoxin B1 is metabolized into aflatoxin M1 [67]. Aflatoxin M1 is then excreted into milk during lactation [65]. Residual aflatoxins may also accumulate in edible animal tissues such as liver and muscle, and may be excreted in urine [68]. Humans are exposed when they consume these contaminated animal products, especially milk containing aflatoxin M1 and other edible tissues [64, 68]. This dietary intake represents the main pathway through which aflatoxins enter the human body from animal sources [66].

## Exposure to AF through dermal contact

Although ingestion of contaminated foods is the primary route of aflatoxin exposure, occupational exposure may also contribute significantly in certain work environments [66]. Individuals working in food processing facilities, livestock feed industries, storage warehouses, and grain distribution centers may be exposed to aflatoxins through inhalation of contaminated dust [66, 69]. They may also experience direct dermal and hand–surface contact with contaminated raw materials, equipment, or surfaces, particularly where occupational safety measures and personal protective equipment are inadequate. In such high-risk occupational settings, airborne aflatoxin-contaminated particulates may lead to additional exposure beyond levels typically encountered through dietary intake [70].

### Exposure to AF through inhalation

Aflatoxin exposure occurs through multiple environmental and biological pathways rather than being restricted to contaminated food alone [71]. Environmental and occupational exposure primarily arises from inhalation of airborne particles such as fungal spores, fragmented hyphae, and dust. These particles may carry aflatoxins into the respiratory tract [72]. Upon inhalation, they first interact with the nasal cavity and airway epithelium, which act as initial biological barriers before deeper pulmonary penetration [73, 74]. In addition, inadequate workplace ventilation and continuous exposure to contaminated environments further increase overall aflatoxin burden. This indicates that exposure pathways extend beyond dietary intake and include significant environmental routes [71].

**Table 3:** Human aflatoxin exposure pathways and supporting evidence

Exposure pathway	Source	Route of entry	Vulnerable population	Biomarkers or indicators	Representative study findings	Implication	References
Contaminated food ingestion	Contaminated maize, groundnuts, cereals, nuts, and improperly stored staple foods colonized by Aspergillus species	Oral ingestion	The general population, particularly populations in Africa and Asia, with poor food storage systems	Dietary aflatoxin intake estimates, AFB1 contamination levels, AF-alb adducts	Estimated daily intake ranged from 0.93–2.45 ng/kg bw in Europe, 3.5–180 ng/kg bw in Africa, and 0.3–53 ng/kg bw in Asia; rural maize consumption may reach 1–2 kg/day.	Primary and most important route of human aflatoxin exposure globally	[37–44]
Prenatal (in utero) exposure	Maternal consumption of aflatoxin-contaminated foods during pregnancy	Placental transfer from mother to fetus	Fetuses, neonates, pregnant women	AF-alb adducts, DNA methylation changes	Prenatal exposure associated with altered DNA methylation patterns and lower neonatal body weight	Indicates exposure begins before birth and may affect long-term development	[45–47]
Breast milk exposure	AFM1 is excreted into breast milk following maternal dietary exposure	Lactational transfer during breastfeeding	Breastfed infants and lactating mothers	AFM1 concentration in breast milk, infant HAZ/WAZ indices	AFM1 was detected in breast milk within 12–24 h after maternal intake; concentrations ranged from 0.01–0.55 ng/mL in Tanzania	Demonstrates continuous infant exposure during breastfeeding	[29, 48–54]
Complementary/weaning food exposure	Contaminated maize- and groundnut-based weaning foods	Oral ingestion during complementary feeding	Infants and children under five years	Serum aflatoxin biomarkers, HAZ, WAZ, WHZ scores	Biomarker levels increased after the introduction of complementary foods and peaked around 3 years of age.	Highlights high susceptibility during the first 1,000 days of life	[55–62]
Animal-source food exposure (carryover exposure)	Milk, meat, eggs, and tissues from livestock fed contaminated feed	Indirect dietary ingestion	Consumers of dairy and livestock products	AFM1 in milk, aflatoxin residues in tissues	Residual aflatoxins may persist in milk and edible tissues despite partial detoxification in ruminants.	Important secondary dietary exposure pathway	[63–66]
Occupational dermal exposure	Contaminated grains, feed, storage dust, industrial surfaces, and handling equipment	Skin contact	Agricultural workers, feed handlers, grain storage workers, food processing workers	Occupational exposure studies, skin permeability studies	Mycotoxins are capable of penetrating the skin barrier in occupational settings	Occupational safety concern where PPE and hygiene measures are inadequate	[66–70]
Occupational and environmental inhalation exposure	Airborne fungal spores, contaminated dust particles, and fragmented fungal hyphae	Inhalation through the respiratory tract	Workers in warehouses, feed industries, grain mills, and contaminated indoor environments	Airborne aflatoxin measurements, respiratory exposure studies	Inadequate ventilation significantly increases the risk of airborne aflatoxin exposure	Important but under-recognized non-dietary exposure route	[71–74]

## Health effects of aflatoxin exposure

**Fig. 2:**
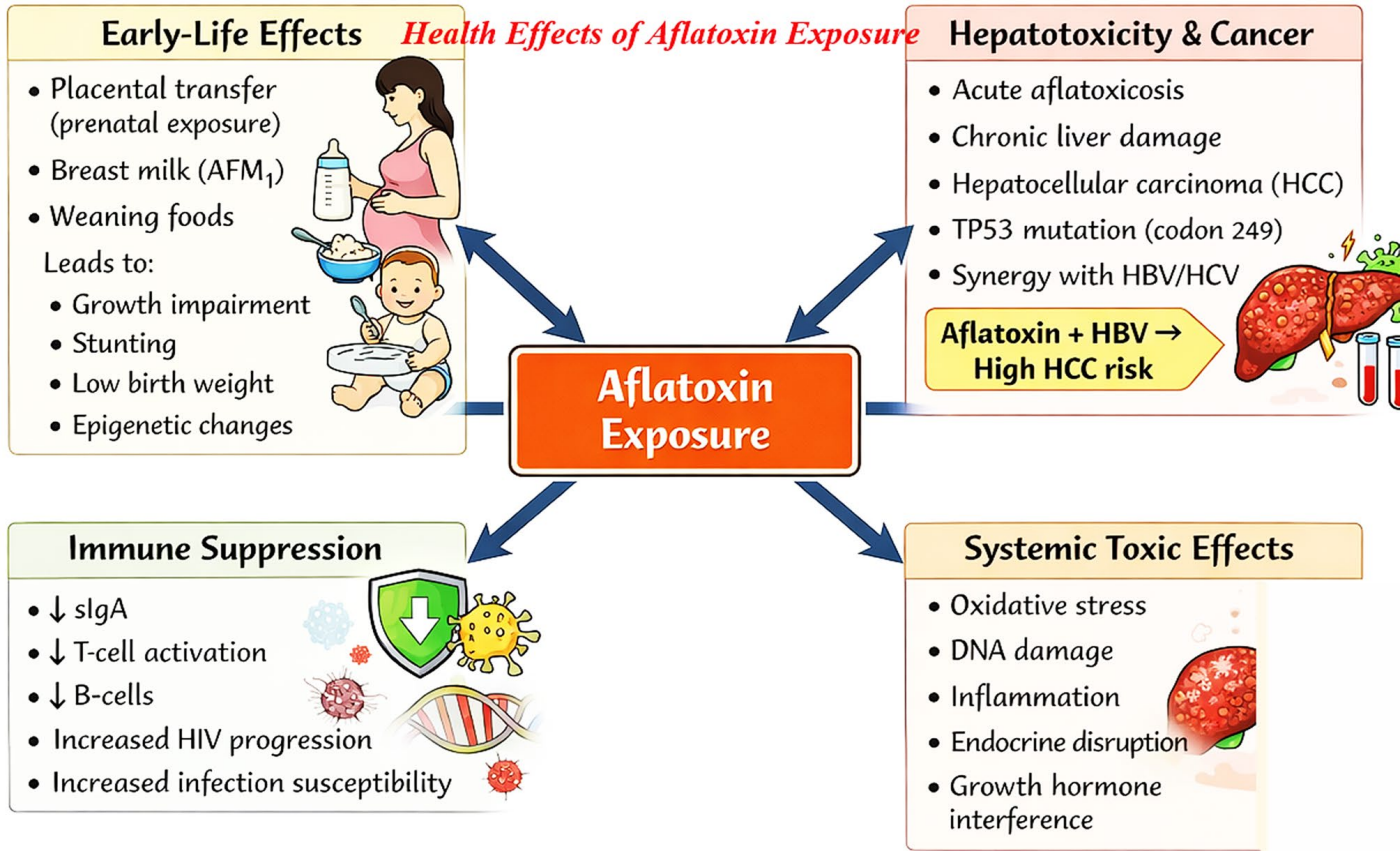
Health effects of aflatoxin exposure

Dietary aflatoxin exposure is associated with multiple adverse health outcomes across different stages of life. Prenatal exposure has been linked to impaired fetal development [63]. In infancy, aflatoxin-related metabolites have been associated with an early-life toxic burden, with potential implications for growth and development [52]. In early childhood, aflatoxin exposure is strongly associated with impaired physical growth, including reduced linear growth and poor nutritional status during critical developmental periods [77]. High-level exposure can lead to acute aflatoxicosis, a severe toxic condition characterized by markedly elevated AF-alb adduct concentrations compared with non-exposed individuals [9]. Chronic low-dose exposure is more common globally and is strongly associated with long-term health consequences. Households that obtained maize through market purchases were 3.64 times more likely to experience elevated aflatoxin levels [78]. The most important chronic outcome is HCC, which represents the primary aflatoxin-related malignancy [79]. Additional long-term effects include impaired childhood growth and suppression of immune function [80]. These effects contribute substantially to increased morbidity and disease susceptibility in affected populations (Table 4; Fig. 2).

### Impaired child growth

The WHO defines stunting as a HAZ below − 2, underweight as a WAZ below − 2, and wasting as a WHZ below − 2 [81]. The impact of aflatoxin exposure on physical development throughout different life stages has been investigated in several studies [81]. Prenatal aflatoxin exposure may contribute to reduced neonatal body weight [75] and impaired linear growth during childhood [76]. Exposure to aflatoxin M1 (AFM1) through breast milk has been associated with reduced height-for-age z-scores (HAZ) and weight-for-age z-scores (WAZ) in infants [52]. Exposure through contaminated weaning foods has consistently been linked to stunting, wasting, and underweight conditions in children across multiple African and Asian populations [60, 62]. These findings suggest that aflatoxin exposure during critical developmental stages may impair normal growth and nutritional status in children.

## Hepatocellular carcinoma

South Asia and Sub-Saharan Africa bear the highest global burden of liver cancer, mainly driven by long-term dietary exposure to aflatoxins through contaminated food consumption [82]. It is estimated that nearly 250,000 deaths in China and Africa are associated with HCC [83]. Worldwide, aflatoxin exposure accounts for approximately 4.6%–28.2% of HCC cases, with mortality reaching about 25% in highly exposed populations. Individuals at highest risk include children and patients chronically infected with HBV or hepatitis C virus (HCV), because viral infections substantially increase susceptibility to aflatoxin-related carcinogenesis [84].

In Ethiopia, persistent aflatoxin exposure has been associated with an estimated 11–288 HCC cases, while co-exposure to aflatoxins and HBV increases this burden to 21–643 cases, reflecting the country’s HBV prevalence of 6–7% [85]. This contrast indicates that aflatoxin-related carcinogenic risk is not uniformly distributed but is highly context-dependent, driven by environmental exposure and infectious disease prevalence. By 2012, liver cancer ranked as the sixth most frequently diagnosed cancer globally, with 83% of cases occurring in low- and middle-income countries [86]. The highest incidence rates are reported in African and Asian countries, where aflatoxins combined with HBV and HCV infections represent major etiological factors [87]. These findings show a consistent global-to-local pattern where aflatoxin exposure acts synergistically with viral hepatitis to amplify HCC burden, particularly in resource-limited settings.

A strong synergistic interaction exists between aflatoxin exposure and HBV infection, substantially amplifying HCC risk [88]. A well-established molecular biomarker of aflatoxin exposure is mutation of the TP53 gene at codon 249 [89]. Villar et al. (2011) identified seasonal variation in the R249S mutation in circulating cell-free DNA among Gambian participants [90]. This variation corresponded with changes in aflatoxin exposure and HBV prevalence, thereby confirming the interactive role of these factors in liver carcinogenesis. A systematic review and meta-analysis including 17 case-control and cohort studies from sub-Saharan Africa, China, and Taiwan estimated the population attributable risk (PAR) of aflatoxin-associated HCC [91]. Aflatoxin exposure contributed to 17% of HCC cases, with higher attribution among HBV-positive individuals (21%) than HBV-negative populations (8.8%) [91]. This indicates a strong synergistic effect between aflatoxin exposure and HBV infection, with markedly increased HCC risk in co-exposed populations.

In China, public health interventions introduced during the 1980s were associated with substantial reductions in primary liver cancer incidence [92]. These interventions included a dietary transition from maize-based diets to rice-based diets with lower aflatoxin contamination, as well as nationwide HBV vaccination. Data from the Qidong Cancer Registry were combined with serum AF-alb measurements collected from seven separate cohorts between 1982 and 2012 [54]. These data were used to estimate a population-attributable fraction; the reduction in primary liver cancer mortality linked to these changes was approximately 65%. This decline indicates a strong population-level impact of long-term aflatoxin control measures, linking reduced exposure to measurable reductions in hepatocellular carcinoma mortality. AF-alb concentrations declined from 19.3 pg/mg in 1989 to undetectable levels by 2012, confirming the effectiveness of integrated prevention strategies [9]. This marked reduction in biomarker levels provides direct biological evidence of successful exposure mitigation at the community level.

Risk assessment studies evaluating AFM1 exposure from dairy products generally reported very low HCC incidence [31]. Alameri et al. (2023) identified pasteurized and ultra-high-temperature (UHT) milk as the major dietary sources of AFM1 exposure and estimated annual HCC incidences of 0.00038 and 0.00039 cases per 100,000 individuals under lower- and upper-bound exposure scenarios, respectively [93]. Children aged 1–3 years showed the highest estimated risk (0.00034 cases per 100,000), although overall exposure was considered negligible from a public health perspective [93]. These findings indicate that current exposure levels are extremely low and unlikely to meaningfully contribute to population-level hepatocellular carcinoma burden. However, AFM1 contamination in commonly consumed dairy products still represents a continuous low-dose exposure pathway.

Similarly, Conteçotto et al. (2021) evaluated AFM1 exposure from UHT milk, powdered milk, and infant formula and estimated HCC risks ranging from 0.0015 to 0.0045 cases per 100,000 children, with average incidences of 0.0027–0.0029 per 100,000 [94]. Torović et al. (2021) also reported very low AFM1-related risk among Malawian households consuming raw milk, with projections of 0.038 and 0.023 cases per 100,000 for children and adults, respectively [95]. Both studies consistently indicate that AFM1 exposure through milk and milk-based products is associated with very low but non-zero estimated HCC risk across different populations and food systems. However, the magnitude of aflatoxin exposure and the dietary sources differ considerably between regions. Conteçotto et al. focused on processed dairy products (UHT milk, powdered milk, and infant formula) under controlled assumptions. In contrast, Torović et al. assessed raw milk consumption in household settings. This suggests that consumption practices may influence exposure variability. This indicates a gap in evidence linking modeled exposure scenarios to real-world dietary practices and actual risk variability.

Morales-Moo et al. (2020) identified aflatoxin-contaminated popcorn as a measurable contributor to HCC risk [96]. Higher estimated incidence was observed among females (0.993 cases per 100,000 annually). Lower incidences were reported among males and boys under 18 years (0.137 cases per 100,000 annually) [96]. Poormohammadi et al. (2021) assessed aflatoxin B2 (AFB2) exposure from edible oils in Iran and reported carcinogenic risk levels in both adults and children [97]. Exposure was slightly lower in children due to reduced consumption. Comparatively, both studies confirm that dietary aflatoxin exposure contributes to measurable cancer risk. However, they differ in exposure sources, with Morales-Moo et al. focusing on popcorn and Poormohammadi et al. on edible oils. They also differ in exposure compounds, involving mixed aflatoxins versus AFB2. In addition, population patterns vary, with Morales-Moo et al. reporting a higher incidence in females, while Poormohammadi et al. observed a more uniform risk across adults and children.

Dietary AFB1 significantly increases the risk of cirrhosis and liver cancer, especially among individuals with chronic HBV infection, further demonstrating the strong toxin–virus interaction [98]. Li et al. (2025) reported that children in North-Central Nigeria had approximately twice the liver cancer risk of adolescents and nearly four times that of adults because of AFB1 contamination in cereals, nuts, and legumes [99]. Saad-Hussein et al. (2014) documented carcinogenic outcomes among wheat-processing workers chronically exposed to aflatoxins, highlighting occupational health risks [100]. These findings indicate that aflatoxin-related carcinogenic risk affects both vulnerable children (mainly through dietary exposure) and occupationally exposed adults (through inhalation and handling in workplaces), but via different exposure pathways.

## Immune suppression

The immunotoxic effects of aflatoxins have been widely demonstrated in experimental animal studies, where exposure results in reduced antibody synthesis, impaired cellular immune responses, and increased susceptibility to infectious diseases [101]. However, evidence in human populations remains comparatively limited, and the full extent of aflatoxin-related immune impairment in humans is not yet fully understood. In The Gambia, a study involving children aged 6–9 years (*n* = 432 with detectable aflatoxin–albumin adducts; *n* = 32 without detectable exposure) reported significantly lower concentrations of secretory immunoglobulin A (sIgA) among exposed children [60]. Because sIgA plays a central role in mucosal defense and pathogen neutralization, reduced levels may weaken barrier immunity and potentially contribute to impaired growth observed in these children. These findings extend previous evidence on aflatoxin-associated growth impairment by introducing an immunological mechanism linking exposure to reduced mucosal immune protection, rather than growth deficits alone. Unlike earlier studies focused mainly on anthropometric outcomes (HAZ, WAZ, and WHZ), this study suggests that immune suppression may be an additional biological pathway mediating or worsening growth faltering. Concurrent exposure to aflatoxin and HBV is especially concerning because their combined effects substantially elevate the risk of HCC compared with aflatoxin exposure alone [89]. These findings suggest not only an association with infection status but also a potential interaction between aflatoxin exposure and viral hepatitis in shaping long-term carcinogenic risk.

In a cohort of 64 adults in Ghana, individuals with higher aflatoxin–albumin adduct concentrations had significantly reduced proportions of activated T lymphocytes (CD3 + CD69+) and activated B cells (CD19 + CD69+) [102]. They also showed decreased CD8 + cytotoxic T cells expressing perforin and/or granzyme A. After adjustment for age and immunological variables, inverse associations remained significant for CD3 + CD69+ and CD19 + CD69+ cells, indicating persistent suppression of immune activation. These alterations may weaken adaptive immunity and increase susceptibility to infectious diseases. The findings indicate aflatoxin-associated immunosuppression characterized by reduced T-cell and B-cell activation and impaired cytotoxic effector function.

In sub-Saharan Africa, where aflatoxin contamination and HIV prevalence frequently overlap, aflatoxin-induced immune suppression has been proposed as a factor contributing to HIV disease progression [81]. In Ghana, HIV-positive individuals with higher aflatoxin exposure demonstrated significantly lower frequencies of regulatory CD4 + T cells, naive CD4 + T cells, and B lymphocytes compared with individuals with lower exposure [68]. Negative correlations were also observed between aflatoxin–albumin adduct levels and perforin-positive CD8 + T cells, regulatory T cells, and B cells. Furthermore, higher aflatoxin exposure was associated with increased odds of elevated direct bilirubin concentrations, indicating hepatic dysfunction, as well as higher HIV viral load [80]. These findings indicate a converging immunotoxic and hepatotoxic burden, where aflatoxin exposure may worsen immune depletion and impair viral control in HIV-infected individuals. Compared with earlier evidence focused on general immune suppression and child growth impairment, these results extend the evidence to HIV-specific immunopathology. The Ghana study shows targeted reductions in CD4+, CD8 + perforin-positive cells, and B cells, suggesting a more direct and clinically relevant interaction between aflatoxin exposure and HIV disease progression. Additionally, Keenan et al. (2011) reported that HIV-infected adults in Ghana with higher aflatoxin biomarker concentrations had an increased risk of developing tuberculosis compared with those with lower exposure [103]. This differs from findings in immunocompetent populations, where aflatoxin exposure is mainly associated with subclinical immunosuppression rather than overt opportunistic infections. It suggests that immunocompromised individuals may experience a higher risk of infectious diseases under aflatoxin exposure.

**Table 4:** Health effects of aflatoxin exposure across different population groups and life stages

Health effect category	Specific outcome	Population/life stage	Findings	Modifying factors	Reference
Acute toxicity	Acute aflatoxicosis	General population	Elevated AF-alb levels; maize market exposure increases risk 3.64×	Food source (market vs. home)	[9, 78]
Growth impairment	Stunting, wasting, underweight	Infants & children	Reduced HAZ, WAZ; widespread growth impairment	Nutritional status	[52, 60, 62, 75, 76, 81]
	Reduced birth weight	Neonates	Lower neonatal body weight linked to prenatal exposure	Maternal exposure level	[75]
	Growth faltering	Children	Consistent association with contaminated weaning foods	Repeated exposure	[46, 47]
Hepatocellular carcinoma (HCC)	Liver cancer burden	General population	4.6–28.2% of HCC attributable; ~250,000 deaths	HBV/HCV prevalence	[83, 84]
	Synergistic carcinogenesis	HBV/HCV-infected individuals	Strong increase in HCC risk with co-exposure	Viral infection status	[84, 88]
	Ethiopia HCC burden	General population	11–288 cases (AFB1), 21–643 with HBV co-exposure	HBV prevalence	[85]
	TP53 mutation (R249S)	Exposed populations	Mutation strongly linked to aflatoxin exposure	HBV infection	[89, 90]
	Population attributable risk	Multi-country populations	17% HCC attributable; higher in HBV+ (21%)	Viral infection	[91]
	Prevention impact (China)	General population	65% reduction in HCC mortality after interventions	Diet change, vaccination	[9, 54, 92]
	AFM1 milk risk	Children	Extremely low HCC risk (0.00038–0.00039/100,000)	Age (1–3 years highest)	[93]
	Processed dairy exposure	Children	Very low risk (0.0015–0.0045/100,000)	Processing level	[94]
	Raw milk exposure	Children & adults	Low incidence (0.023–0.038/100,000)	Household practices	[95]
	Food-specific exposure	Females, children	Popcorn: up to 0.993/100,000; oils are also carcinogenic	Gender, diet type	[96, 97]
	Occupational exposure	Workers	Increased cancer outcomes in wheat processing workers	Workplace safety	[100]
	Childhood dietary exposure	Children	2×–4× higher risk vs. adults/adolescents	Age	[99]
	Liver disease progression	HBV-infected individuals	Increased cirrhosis and HCC risk	Viral load	[98]
Immune suppression	Reduced mucosal immunity (sIgA)	Children (6–9 years)	Lower sIgA weakens mucosal defense	Nutritional status	[60]
	T- and B-cell suppression	Adults	Reduced CD3 + CD69+, CD19 + CD69+, CD8 + function	Exposure intensity	[102]
	HIV progression	HIV-positive individuals	Lower CD4+, CD8+, increased viral load	HIV status	[68, 80]
	Tuberculosis risk	HIV-infected adults	Higher TB risk with higher exposure	Co-infections	[103]
	General immunotoxicity	Animal models	Reduced antibody production and immune response	Dose-dependent exposure	[101]
	Infection susceptibility	Humans	Increased vulnerability to infections	Nutritional status	[81]

## Detection technologies

**Fig. 3:**
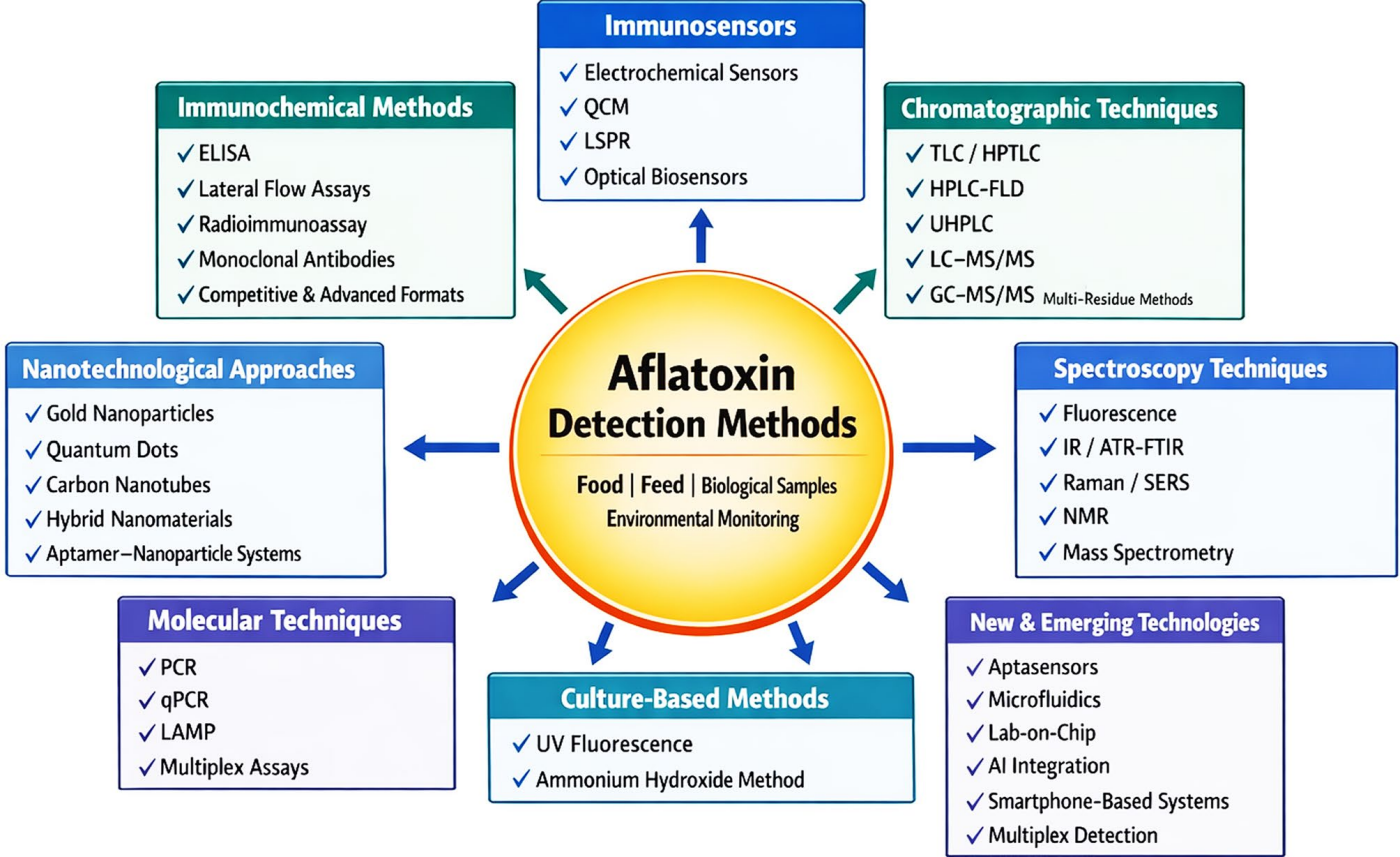
Integrated analytical framework for aflatoxin detection

Aflatoxins are commonly detected based on their characteristic optical properties, including a distinct absorption maximum near 360 nm and their molecular structure [104]. These physicochemical features underpin a range of analytical approaches used for surveillance and control in food and feed systems. Since their discovery in the 1960s [4], detection strategies have evolved from early microbiological culture-based methods to more advanced analytical platforms with improved sensitivity and specificity. Modern surveillance systems increasingly rely on high-performance liquid chromatography (HPLC), enzyme-linked immunosorbent assays (ELISA), fluorescence-based techniques, and other spectroscopic methods as key analytical tools for aflatoxin monitoring [1, 4], which represent major categories within aflatoxin surveillance frameworks (Fig. 3).

### Chromatographic techniques

Over the years, several analytical platforms have been developed for the determination of aflatoxins in food and foodstuff samples [105]. Early methods, such as thin-layer chromatography (TLC), were widely applied but showed limitations in environmental stability and analytical precision, leading to their replacement by more advanced techniques. Contemporary approaches mainly rely on HPLC coupled with fluorescence detection (FLD) or ultraviolet detection at 360 nm, following appropriate extraction and purification procedures [106]. Among the available analytical techniques, HPLC-FLD offers high sensitivity and specificity, making it a widely adopted method for aflatoxin analysis. HPLC-based methods represent the core analytical backbone for routine aflatoxin monitoring due to their balance of sensitivity, reproducibility, and regulatory acceptance, although they require significant laboratory infrastructure. At the biomonitoring level, differences in aflatoxin biomarkers are also observed across biological matrices, according to the period of exposure assessed. Blood-based samples, such as plasma and serum, and excretory samples, such as urine, capture different exposure timeframes and vary in the stability of metabolites, which affects the comparability of analytical results between studies [107].

Advanced planar chromatography, particularly high-performance thin-layer chromatography (HPTLC), offers improved resolution and quantitative accuracy compared with conventional TLC, with validated detection limits reaching 0.5 ppb. Standard HPLC systems, including pre-column and post-column derivatization strategies, are officially accepted for regulatory aflatoxin analysis and allow flexible analytical configurations depending on the analyte and matrix [108]. Liquid chromatography–tandem mass spectrometry (LC–MS/MS) represents a highly sensitive and selective analytical platform for aflatoxin detection. It can simultaneously identify the four major aflatoxins with detection limits below 1 ng/mL [109]. It is widely used for quantification and biomonitoring in biological matrices such as plasma, serum, and urine. The method also enables multi-analyte detection, including AFB1, AFM1, AFQ1, and AFP1 [109]. Among chromatographic techniques, LC–MS/MS is considered best-in-class due to its ultra-trace sensitivity, multiplex detection capability, and suitability for biomonitoring applications. However, it is cost-intensive and requires advanced technical expertise. Conversely, despite its analytical superiority, biomarker expression and metabolic conversion rates vary across individuals. This variability can influence quantitative interpretation in epidemiological studies. It highlights the need for standardized biomonitoring frameworks.

TLC separates aflatoxins through differential adsorption on a stationary phase, using various solvent systems such as acetic acid, acetone, acetonitrile, chloroform, peroxide-free ether, ethanol, hexane, isopropanol, and toluene to optimize migration [110]. In contrast, HPLC provides precise analytical control through adjustments in mobile phase composition, gradient programs, detection systems, and column characteristics, including normal-phase and reversed-phase configurations. Reversed-phase columns with acidic mobile phases composed of acetonitrile, methanol, water, or their combinations are most commonly used [111]. Gas chromatography (GC) separates compounds according to their volatility and retention time. However, aflatoxins are relatively polar, non-volatile, and thermally unstable, making them unsuitable for direct GC analysis without derivatization [112]. Therefore, GC is not routinely used for aflatoxin determination. In comparative terms, TLC serves mainly as a low-cost screening tool, HPLC as a standard quantitative method, and GC is of limited applicability for aflatoxins due to physicochemical constraints.

Recent advances combining ultra-performance liquid chromatography (UPLC) with triple quadrupole mass spectrometry (QqQ) have significantly enhanced analytical speed, precision, and sensitivity for aflatoxin analysis. LC–MS/MS is currently regarded as the gold-standard method for biomonitoring studies because of its picogram-level sensitivity and ability to minimize matrix interferences [113]. UPLC–MS/MS further improves throughput and resolution, making it the most advanced chromatographic platform for high-throughput analytical environments. Comparative evaluation indicates a distinct ranking of analytical methods based on performance. LC–MS/MS and UHPLC–MS provide superior sensitivity and analytical reliability, while HPLC and HPTLC are practical mid-level techniques suitable for routine laboratory use. TLC and GC, in contrast, are mainly limited to preliminary screening or specific, specialized applications [114].

Shi et al. (2026) developed a rapid HPTLC method for detecting AFB1 in millet and buckwheat and DON in cereals, using acetonitrile extraction and smartphone-assisted quantification, with detection limits of 14.2 µg/kg for AFB1 [115]. Nguyen et al. (2023) optimized HPTLC and UPLC-FLD methods for simultaneous aflatoxin quantification in peanuts and raisins, reporting detection limits between 0.008 and 0.1 µg/kg, recovery rates from 76.5% to 99.8%, and identifying AFB1 in 28.6% of peanut samples [116]. These studies demonstrate that planar chromatography remains useful for screening, while LC-based techniques dominate quantitative accuracy.

Muñoz-Solano et al. (2020) established an LC-FLD method for aflatoxin determination in animal feed, achieving detection limits between 0.64 and 2 µg/kg with recovery values from 73.6% to 88.0% [117]. Khabbouchi et al. (2024) analyzed 1,053 spice samples using HPLC–MS/MS and reported aflatoxin contamination in 2.3% of samples, with the highest concentrations in bay leaves and chili powder [118]. Abdel-Azeem et al. (2015) applied UHPLC combined with solid-phase extraction (SPE) for aflatoxin monitoring in food products, obtaining recoveries between 89.6% and 103.3% and detection limits of 0.02–0.04 µg/kg [119]. Rodríguez-Carrasco et al. (2012) used GC–MS/MS for simultaneous identification of ten mycotoxins in wheat semolina, achieving recovery rates ranging from 74% to 124% [120]. UHPLC and LC–MS/MS demonstrate the highest analytical robustness, while GC–MS/MS is mainly applicable for multi-mycotoxin profiling rather than routine aflatoxin monitoring.

Chromatographic and analytical approaches serve complementary purposes in identifying aflatoxins. LC–MS/MS and UHPLC–MS/MS are regarded as definitive, high-precision techniques because of their extremely low detection limits and their applicability in biomonitoring studies. In contrast, HPLC platforms are widely used for routine quantitative testing in laboratories, offering reliable measurement supported by established standardization and compliance with regulatory requirements [121]. Conversely, TLC and HPTLC are low-cost and rapid screening techniques. They are mainly used for preliminary assessment, especially in resource-limited settings. In contrast, gas chromatography–tandem mass spectrometry (GC–MS/MS) is mainly used for multi-mycotoxin profiling rather than routine aflatoxin-specific analysis [122]. This complementary framework ensures a balance between analytical sensitivity, speed, cost-effectiveness, and field applicability across different stages of aflatoxin detection and risk assessment.

### Spectroscopy techniques

Spectroscopic techniques are essential analytical tools that investigate interactions between electromagnetic radiation and chemical substances across a broad spectral range, from radiofrequency to gamma radiation [123]. Depending on the wavelength, these methods characterize distinct molecular properties. When radiation interacts with compounds, it may be absorbed or emitted; absorption-based techniques measure decreases in light intensity, whereas emission-based methods quantify radiation released from excited molecules [124]. In general, spectroscopy provides rapid, often non-destructive analysis, but varies widely in sensitivity depending on the technique used.

Mass spectrometry is among the most accurate and sensitive platforms for aflatoxin determination, enabling both identification and precise quantification in complex food and biological matrices [125]. The analytical workflow includes sample preparation, chromatographic separation, ionization, mass-to-charge analysis, and data interpretation. Sample preparation is a critical step and may represent the majority of the process [126]. Common extraction and cleanup methods include solvent-based extraction, SPE, liquid–liquid extraction (LLE), liquid–solid extraction (LSE), and QuEChERS techniques, selected according to sample type and analytical needs [127]. Magnetic solid-phase extraction has shown efficient recovery of AFB1 and strong reproducibility when coupled with HPLC–MS/MS analysis [128, 129]. Among spectrometric techniques, mass spectrometry is the most sensitive and reliable platform, especially when integrated with chromatographic separation, but it is highly dependent on complex sample preparation.

Nuclear magnetic resonance (NMR) spectroscopy complements mass spectrometry by utilizing the magnetic properties of nuclei such as 1 H and 13 C [130, 131]. It provides detailed structural information, including functional groups, connectivity, and stereochemistry of aflatoxins. NMR also supports metabolomic studies of aflatoxin biotransformation and detoxification pathways [132], and contributes to laboratory quality assurance, reproducibility, and structural accuracy [133]. NMR is therefore considered the best technique for structural elucidation, while MS dominates quantitative trace detection. Fluorescence-based detection exploits the intrinsic fluorescent properties of aflatoxins. Upon excitation at specific wavelengths, these compounds emit measurable signals proportional to concentration [134, 135]. Infrared (IR) spectroscopy detects aflatoxins by monitoring vibrational transitions of functional groups induced by infrared radiation. Characteristic absorption bands related to carbonyl, aromatic, and alkene structures enable identification [136]. Fluorescence spectroscopy is the most practical for rapid screening, while IR provides complementary structural fingerprinting with lower sensitivity.

Dhanshetty et al. (2022) developed a multi-residue method for simultaneous determination of aflatoxins B1, B2, G1, and G2 in chili powder using methanol–water extraction, immunoaffinity column purification, UHPLC-FLD, and LC–MS/MS [137]. Li et al. (2025) reported that integrating NMR with mass spectrometry enhances structural elucidation and detection accuracy, particularly using high-field magnets and cryogenic probes to improve spectral resolution [138]. These innovations show a trend toward hybrid analytical systems combining spectroscopy and portable technologies for field-based detection.

Sein et al. (2024) applied ATR–FTIR spectroscopy combined with machine learning for AFB1 detection in chili powder [139]. Deng et al. (2022) optimized Raman spectroscopy using BOSS, VCPA, and CARS feature-selection methods for AFB1 quantification in maize [140]. Chemometric-enhanced spectroscopy significantly improves predictive accuracy, compensating for intrinsic sensitivity limitations of vibrational techniques. Importantly, variability in aflatoxin biomonitoring is influenced by both analytical and biological factors, including matrix interference, metabolic differences, and individual exposure variation, particularly in AF–albumin adducts and urinary metabolites [141]. Reliability varies by method, with LC–MS/MS and UHPLC giving the most consistent and reproducible results, while fluorescence and infrared techniques show lower accuracy and greater variability [142]. In analytical performance comparison, mass spectrometry serves as the gold standard for quantification, NMR is preferred for structural elucidation, and spectroscopy is mainly used for rapid screening with lower sensitivity [143]. The suitability of each technique depends on its use case, where mass spectrometry is primarily applied for definitive laboratory identification and exposure monitoring, whereas fluorescence-based methods, infrared spectroscopy, and portable analytical devices are mainly utilized for on-site screening and routine field surveillance [144].

These analytical platforms play complementary roles. Chromatography coupled with mass spectrometry, particularly LC–MS/MS and UHPLC–MS/MS, is widely used as highly sensitive reference and confirmatory methods for regulatory analysis [145]. In contrast, spectroscopic approaches and biosensor-based technologies provide fast, economical, and portable options for on-site screening, early identification, and monitoring purposes. This integrated approach enhances both analytical accuracy and practical applicability across laboratory and field settings.

## Biosensors (immunosensors + nanobiosensors + molecular sensing platforms)

The identification of mycotoxins, particularly aflatoxins, is primarily based on immunological assays that rely on specific antigen–antibody recognition [146]. Because aflatoxins are low-molecular-weight compounds, they must be chemically conjugated to larger carrier proteins to generate immunogenic complexes capable of stimulating antibody production. Among immunoassay techniques, ELISA, especially the competitive format, is the most widely applied screening method for aflatoxin detection in liquid matrices, dairy products, agricultural commodities, and processed foods [147]. ELISA remains the foundational biosensing platform, offering high specificity and reliability but limited field portability compared to newer systems. Differences in ELISA performance are largely driven by interference from sample matrices and variations in the effectiveness of antigen–antibody binding [148].

To enhance analytical performance, several modified ELISA-based and advanced immunoassay platforms have been developed. Lateral flow assays (LFAs) are widely used for rapid field-based aflatoxin screening due to their portability, low cost, and ease of operation, although their sensitivity is generally lower than that of ELISA [149]. LFAs are the most feasible biosensor platform for point-of-care use due to their ease of operation and rapid results. However, this practicality comes at the cost of reduced analytical sensitivity. Compared with other immunoassay techniques, LFAs offer the greatest suitability for field application but exhibit the lowest detection sensitivity [150].

Immunosensing platforms integrate antibody-based recognition with advanced transducer systems to enable rapid, highly sensitive, and real-time detection of aflatoxins [151]. These approaches include ELISA, electrochemical immunosensors, lateral flow devices, gold-based immunochromatographic tests, and aptamer-integrated formats [152]. They outperform optical and colorimetric systems in sensitivity but show variable reproducibility in complex matrices. Azri et al. (2018) developed an indirect competitive differential pulse voltammetry (DPV) immunosensor using a multi-walled carbon nanotube/chitosan (MWCNTs/CS)-modified screen-printed carbon electrode (SPCE), achieving a calibration range of 0.0001–10 ng/mL and a limit of detection (LOD) of 0.3 pg/mL [153]. Electrochemical immunosensors represent the highest-performing biosensor class in terms of analytical sensitivity and ultra-trace detection capability.

Karachaliou et al. (2022) highlighted wavelength-resolved label-free (WLRS) immunosensing systems as highly selective, real-time platforms suitable for point-of-care applications [154]. Among optical biosensors, LSPR and WLRS platforms provide the best balance of speed, sensitivity, and real-time monitoring capability. Optical-based detection systems act as bridging technologies positioned between highly sensitive electrochemical sensing platforms and inexpensive lateral flow assays (LFAs) [150].

Nanotechnology offers powerful platforms for the identification of aflatoxins, including gold nanoparticles (AuNPs), carbon-based nanomaterials (CBNs), magnetic nanoparticles (MNPs), quantum dots (QDs), upconversion nanoparticles (UCNPs), and hybrid nanostructures [155]. Nanobiosensors include colorimetric, fluorescence, electrochemical, and nanoparticle-integrated systems [156]. Nanobiosensors represent the most advanced biosensing frontier due to signal amplification, miniaturization, and multiplexing potential [157]. Nanobiosensors offer very strong signal enhancement capabilities; however, their performance can be inconsistent due to issues with stability and reproducibility.

A variety of DNA-based diagnostic tools, including PCR, LAMP, and qPCR, are widely used for detecting genetic markers associated with aflatoxin biosynthesis in fungal species [158]. However, they have limited field applicability compared to immunoassays. Kumsiri et al. (2020) demonstrated that qPCR and qLAMP showed the highest sensitivity (5 pg) compared with conventional PCR and LAMP [159]. Quantitative molecular techniques such as qPCR and qLAMP are presently regarded as the most highly sensitive nucleic acid–based methods for the early detection of aflatoxin-producing fungal species [159]. Historically, phenotypic screening techniques such as UV fluorescence detection and ammonium hydroxide vapor methods have been applied for identifying aflatoxin-producing fungi [160]. These methods remain useful as low-cost preliminary screening tools but are limited by subjectivity and low analytical precision. They are the least reliable but most cost-effective methods.

These detection approaches serve complementary roles within a tiered analytical framework. High-sensitivity laboratory-based methods such as LC–MS/MS, advanced immunosensors, and qPCR/qLAMP function as confirmatory or reference techniques for precise quantification and validation. In contrast, ELISA, LFAs, and phenotypic methods provide rapid, low-cost, and field-deployable screening tools for early detection and surveillance [161]. Nanotechnology-enhanced and optical biosensors bridge these two levels by improving sensitivity while maintaining portability, thereby linking screening and confirmatory diagnostics within integrated aflatoxin monitoring systems [162].

### Newer and emerging approaches for aflatoxin detection

Recent developments in aflatoxin detection emphasize molecular recognition systems, nanomaterials, and miniaturized analytical platforms to improve sensitivity, selectivity, speed, and field applicability [161]. These approaches are especially useful where LC–MS/MS is limited by cost and infrastructure requirements. Although LC–MS/MS remains the reference standard for confirmatory quantification and regulatory analysis due to its high accuracy, multiplex capability, and strong analytical reliability, emerging technologies provide rapid and portable alternatives for decentralized screening [163]. These include biosensors, surface-enhanced Raman scattering (SERS), microfluidic devices, multiplex immunoassays, and lab-on-a-chip systems. Such technologies represent a tiered analytical ecosystem rather than isolated methods. In this context, biomonitoring markers also show inherent variability in sensitivity, biological half-life, and matrix-dependent performance, which influences their reliability across different exposure and population settings.

Across these technologies, performance can be classified into three main tiers [164]. (i) Highly advanced analytical platforms are mainly based on LC–MS/MS and UHPLC–MS systems, which provide very high sensitivity, strong structural confirmation of compounds, and simultaneous detection of multiple analytes in one run [165]. Their main strength is regulatory-grade accuracy and broad applicability across complex matrices, while their limitations include high cost, complex operation, and lack of field deployability. (ii) Advanced biosensing and integrated analytical platforms comprise electrochemical immunoassay sensors, localized surface plasmon resonance (LSPR) optical detection systems, quartz crystal microbalance (QCM) devices, and immunosensors enhanced with nanomaterials [166]. These systems achieve very high sensitivity through signal amplification strategies and nanostructure integration, enabling near real-time detection. Their strengths lie in miniaturization, rapid response, and potential for point-of-care use, while weaknesses include matrix interference, limited long-term stability, and variability in reproducibility. Electrochemical immunosensors and surface-enhanced Raman scattering (SERS)–based platforms are regarded as top-performing technologies in this category [167]. This is because they provide highly efficient signal enhancement and enable detection at extremely low (trace) concentrations. (iii) Quick detection and portable diagnostic technologies consist of lateral flow immunoassays, paper-based microfluidic devices, and nanoparticle-based color change tests [150]. These platforms are advantageous due to simplicity, low cost, and instrument-free operation, but they suffer from lower sensitivity and reduced quantitative precision.

Importantly, these tiers are complementary rather than competitive. Advanced chromatographic techniques and mass spectrometry–based analytical systems function as definitive confirmatory methods, whereas mid-level biosensor technologies act as a link between laboratory-based analysis and on-site testing by enabling fast, semi-quantitative detection [168]. Quick detection methods support initial monitoring and provide early alert systems, especially in low-resource or field environments [169]. This layered workflow supports stepwise decision-making from initial screening to definitive confirmation.

Aptamer-based biosensors provide a stable and highly specific alternative to antibody-based systems with improved reproducibility and flexible signal transduction [170]. Paper-based microfluidic platforms with aptamer-functionalized nanoparticles enable simple colorimetric detection and low-cost deployment [171]. SERS-based systems enhance molecular signals via plasmonic nanostructures, enabling ultra-trace detection with rapid response and high specificity [172]. Integrated microfluidic–nanomaterial immunosensors (e.g., PDMS extraction with carbon nanotubes) reduce sample volume while maintaining regulatory-level sensitivity and enabling full sample-to-result workflows [173]. These represent convergent hybrid platforms combining recognition, amplification, and miniaturization.

Multiplex immunoassays and cytometric bead-based systems enable simultaneous detection of multiple mycotoxins in a single run, improving throughput and surveillance efficiency [174]. LC–MS/MS remains essential for confirmatory analysis and regulatory compliance due to its unmatched analytical certainty, despite cost and operational complexity [175]. Therefore, LC–MS/MS serves as the primary reference standard within the hierarchy of analytical methods, acting as the benchmark against which other techniques are validated [176]. Aflatoxin detection is evolving toward integrated hybrid systems combining aptamer engineering, nanomaterials, SERS enhancement, and microfluidics [177]. These convergent platforms aim to balance analytical accuracy with operational practicality by enabling rapid, portable, scalable, and highly sensitive detection suitable for both laboratory confirmation and field screening.

## Challenges of aflatoxin detection technologies

Despite significant advances in rapid and portable diagnostic tools, many existing methods still lack the analytical sensitivity required for reliable trace detection at internationally regulated parts-per-billion (ppb) levels. Because aflatoxins are highly toxic even at extremely low concentrations, insufficient sensitivity in some field kits and point-of-care assays may compromise food safety monitoring. Analytical performance is further affected by complex food and feed matrices. Naturally occurring components such as lipids, pigments, phenolic compounds, and other interfering substances can reduce specificity and signal accuracy in immunological, optical, and chromatographic platforms. These matrix effects are particularly challenging in cereals, nuts, spices, dairy products, and medicinal plants, where validation and reproducibility are demanding.

High-resolution techniques such as HPLC and LC–MS/MS often require extensive pre-analytical procedures, including extraction, purification, and concentration. Errors during sample preparation may lead to analyte degradation or incomplete recovery. In addition, antibody- and aptamer-based assays may exhibit cross-reactivity with structurally related mycotoxins, potentially generating false-positive results. Many detection systems are optimized primarily for AFB1, while simultaneous identification of multiple aflatoxin variants (B1, B2, G1, G2, and M1) remains technically complex.

Advanced analytical instruments require costly equipment, controlled laboratory conditions, and specialized expertise, limiting their accessibility in low-resource regions where contamination is often more prevalent. Moreover, biological recognition elements such as antibodies and enzymes may lose stability under harsh environmental or storage conditions. Field-deployable and nanotechnology-based sensors may also show variability across production batches, affecting standardization and regulatory acceptance. Although hybrid systems can enhance performance, they may increase operational complexity, and many existing methods rely on discrete rather than continuous monitoring, limiting real-time surveillance across the food supply chain.

## Future perspectives in aflatoxin detection technologies

Future strategies should emphasize ultra-sensitive, rapid, affordable, and user-friendly technologies suitable for decentralized and field-based applications. Integration of nanotechnology with biosensor platforms represents a major advancement. Nanomaterials such as gold and silver nanoparticles, quantum dots, carbon nanotubes, and graphene derivatives can enhance signal amplification, reduce detection limits, and support real-time monitoring. Aptamer-based recognition systems are expected to expand further due to their superior thermal stability, high specificity, and reusability compared with traditional antibodies. Their chemical robustness makes them promising alternatives for long-term deployment in diverse environments.

Multifunctional analytical systems that combine immunological recognition with electrochemical, optical, or chromatographic detection can improve robustness and enable simultaneous identification of multiple aflatoxin analogues in complex matrices. Incorporation of microfluidic and lab-on-a-chip technologies can enhance automation, portability, and high-throughput analysis for on-site testing. Portable diagnostic tools, including smartphone-integrated sensors, paper-based analytical devices, and compact electrochemical platforms, may expand access to rapid screening in underserved regions. Integration with cloud-based data systems can enable real-time reporting and surveillance across food distribution networks.

Artificial intelligence and machine learning approaches can further strengthen detection accuracy by analyzing complex signal patterns, improving classification performance, identifying contamination trends, and supporting predictive risk assessment. Additionally, biomimetic receptors and molecularly imprinted polymers (MIPs) offer stable and selective synthetic alternatives to biological recognition elements used in biosensors. Sustainability considerations are increasingly important. Environmentally friendly detection approaches that minimize chemical waste, reduce energy consumption, and limit reagent use align with green analytical chemistry principles. These technological innovations offer significant potential to improve global aflatoxin surveillance systems.

## Limitations of this narrative review

This review adopts a narrative synthesis approach rather than a fully systematic methodology; therefore, study selection was based on relevance and availability rather than predefined eligibility criteria, which may introduce selection bias. Some relevant studies, particularly non-English publications, may not have been included. The review emphasizes recent technological developments and may not comprehensively cover the historical evolution of all detection methods. Furthermore, several emerging technologies discussed here are based on experimental prototypes that may not yet have been validated under real-world field conditions. This work does not include quantitative comparisons such as detection limits, cost analysis, or scalability assessments, limiting direct methodological ranking. Broader contextual aspects, including regulatory harmonization and infrastructure constraints, were not extensively analyzed.

## Conclusion

Aflatoxin contamination is a persistent food safety concern in low- and middle-income countries due to poor storage, climatic conditions, and weak regulation. Staple foods such as maize, groundnuts, and rice are commonly affected, leading to widespread dietary exposure. Exposure also occurs via prenatal transfer, breast milk, and occupational routes, indicating lifelong risk. Biomarker studies confirm widespread internal exposure, but heterogeneity in biomarkers, methods, and exposure windows limits comparability and global burden estimation. Chronic exposure is strongly linked to stunting, immune suppression, and hepatocellular carcinoma, especially in the presence of hepatitis B co-infection. However, the evidence is mainly based on cross-sectional and heterogeneous studies, which limit causal inference. While LC–MS/MS and UHPLC provide high accuracy, their cost limits use in resource-poor settings. Biosensors and rapid tests are more practical but remain insufficiently validated and standardized. Research gaps in aflatoxin exposure assessment include the lack of standardized biomonitoring methods, limited longitudinal evidence, and inadequate validation of field-based diagnostic tools. Future work should focus on harmonized exposure assessment, integration of field screening with confirmatory methods, and longitudinal studies to improve risk quantification and surveillance.

## Supplementary Information

Below is the link to the electronic supplementary material.

Supplementary Material 1

## Data Availability

No data was used for the research described in the article.

## Abbreviations

AFB1: Aflatoxin B1
AFB2: Aflatoxin B2
AFG1: Aflatoxin G1
AFG2: Aflatoxin G2
AFM1: Aflatoxin M1
AF: alb–Aflatoxin–albumin adduct
AFQ1: Aflatoxin Q1
AFP1: Aflatoxin P1
ATR: FTIR–Attenuated Total Reflectance–Fourier Transform Infrared Spectroscopy
AuNPs: Gold Nanoparticles
CBNs: Carbon–Based Nanomaterials
CD3 + CD69+: Activated T lymphocyte marker subset
CD19 + CD69+: Activated B lymphocyte marker subset
CD8+: Cytotoxic T cell marker
CARS: Coherent Anti–Stokes Raman Scattering
CS: Chitosan
DA: Dopaminergic
DNA: Deoxyribonucleic Acid
ELISA: Enzyme–Linked Immunosorbent Assay
FLD: Fluorescence Detection
FTIR: Fourier Transform Infrared
GC: Gas Chromatography
GDP: Gross Domestic Product
HBV: Hepatitis B Virus
HCV: Hepatitis C Virus
HCC: Hepatocellular Carcinoma
HAZ: Height–for–Age Z–score
HIV: Human Immunodeficiency Virus
HPLC: High–Performance Liquid Chromatography
HPTLC: High–Performance Thin–Layer Chromatography
HPV: (if not used, remove; not present in text)
IR: Infrared Spectroscopy
LFA: Lateral Flow Assay
LLE: Liquid–Liquid Extraction
LSE: Liquid–Solid Extraction
LOD: Limit of Detection
LSPR: Localized Surface Plasmon Resonance
MAO-B: Monoamine Oxidase B
MPTP: 1-Methyl-4-Phenyl-1,2,3,6-Tetrahydropyridine
MS: Mass Spectrometry
MWCNTs: Multi-Walled Carbon Nanotubes
NMR: Nuclear Magnetic Resonance
NTDs: Neglected Tropical Diseases
OHS: Occupational Health and Safety
OP: Organophosphate (context-dependent, used in case study section)
OPMD: Oral Potentially Malignant Disorders
PCR: Polymerase Chain Reaction
PD: Parkinson’s Disease
PPE: Personal Protective Equipment
QCM: Quartz Crystal Microbalance
qLAMP: quantitative Loop-Mediated Isothermal Amplification
qPCR: quantitative Polymerase Chain Reaction
QQQ: Triple Quadrupole Mass Spectrometry
RNA-seq: RNA Sequencing
ROS: Reactive Oxygen Species
RDT: Rapid Diagnostic Test
SERS: Surface-Enhanced Raman Scattering
SPE: Solid-Phase Extraction
SPCE: Screen-Printed Carbon Electrode
sIgA: secretory Immunoglobulin A
TLC: Thin-Layer Chromatography
T2DM: Type 2 Diabetes Mellitus
UHPLC: Ultra-High-Performance Liquid Chromatography
UPLC: Ultra-Performance Liquid Chromatography
UV: Ultraviolet
WAZ: Weight-for-Age Z-score
WHZ: Weight-for-Height Z-score
WHO: World Health Organization
