# Supplementary material for: Aflatoxin exposure and health impacts: global burden and advances in detection technologies
Source: Genes Environ. 2026 Jul 31;48:14. doi: 10.1186/s41021-026-00363-1 (PMC13425974; doi:10.1186/s41021-026-00363-1)
Supplement: Supplementary file 1 — Supplementary Material 1 [file 41021_2026_363_MOESM1_ESM.docx]

SANRA—a scale for the quality assessment of narrative review articles

| 1) Justification of the article’s importance for the readership  The importance is not justified ______________________________________________________0  2  The importance is alluded to, but not explicitly justified___________________________________1  The importance is explicitly justified___________________________________________________2  2) Statement of concrete aims or formulations of questions  No aims or questions are formulated________________________________________________0  2  Aims are formulated generally but not concretely or in items of clear questions _____________1  One or more concrete aims or questions are formulated ________________________________2  3) Description of the literature search  The search strategy is not presented ________________________________________________0  2  The literature search is described briefly _____________________________________________1  The literature search is described in detail, including search terms and inclusion criteria _______2  4) Referencing  Key statements are not supported by references _______________________________________0  2  The referencing of key statements is inconsistent ______________________________________1  Key statements are supported by references __________________________________________2  5) Scientific reasoning (e.g., incorporation of appropriate evidence, such as RCTs in clinical medicine)  The article’s point s not based on appropriate arguments ________________________________0  2  Appropriate evidence is introduced selectively ________________________________________1  Appropriate evidence is generally present ___________________________________________ 2  6) Appropriate presentation of data (e.g., absolute vs relative risk; effect sizes without confidence intervals)  Data are presented inadequately ___________________________________________________0  2  Data are often not presented in the most appropriate way_______________________________1  Relevant outcome data are generally presented appropriately ___________________________  12/12 |
| --- |
| Summary |
